# Quantification of Alfacalcidol Tablets Dissolution Content by Chemical Derivatization and LC-MS

**DOI:** 10.1155/2020/6201656

**Published:** 2020-02-07

**Authors:** Yang Liu, Xi Chen, Song Yuan, Wanhui Liu, Lan He, Qingsheng Zhang

**Affiliations:** ^1^National Institutes for Food and Drug Control, Beijing 102629, China; ^2^YanTai University, Shandong 264005, China

## Abstract

Application of liquid chromatography-mass spectrometry (LC-MS) in analyzing the content of alfacalcidol tablets dissolution faces big challenges due to the low amount of alfacalcidol in each tablet and the low ionization efficacy of the compound with electrospray ionization (ESI) or atmospheric-pressure chemical ionization (APCI). Here, extraction, derivatization, and LC-MS quantitation method have been developed and validated for measuring alfacalcidol tablets dissolution content. After alfacalcidol dissolution solution was extracted with dichloromethane to remove surfactant and inorganic salts, alfacalcidol was then derivatized via a Cookson reagent, 4-phenyl-1, 2, 4-triazoline-3, 5-dione (PTAD), under ambient conditions. Alfacalcidol derivative was successfully analyzed by LC-MS. Limit of detection (LOD) of the derivatized alfacalcidol was improved 100 times (0.01 *μ*g/mL) compared with the nontreated compound (1 *μ*g/mL). The new method was then validated following International Conference on Harmonization (ICH) guidance. The method shows a good linearity with *r*^2^ > 0.99. Interday and intraday reproducibility was 3.3% and 7.9%, respectively. This procedure can be used in quantification of alfacalcidol tablets dissolution content and corresponding pharmaceutical quality control.

## 1. Introduction

Alfacalcidol could be converted to calcitriol (1, 25-(OH)_2_D_3)_ in the liver and acts as an important compound for calcium homeostasis, skeleton formation, and differentiation and proliferation of cells [1–3]. Alfacalcidol is mainly used as a therapeutic drug for osteoporosis, rickets, hypocalcemia, hypovitaminosis, chronic renal failure, and hypoparathyroidism in clinical practice [3, 4]. Multiple analytical methods have been reported in analyzing alfacalcidol content. An ultraviolet spectrophotometer (UV) was used in the detection of bulk alfacalcidol active pharmaceutical ingredient (API) [5]. High-performance liquid chromatography (HPLC) was deployed in testing alfacalcidol-related substances [6] or content [7]. Liquid chromatography-mass spectrometry (LC-MS) was applied to determine alfacalcidol content in human or rat plasma [3, 4]. Dissolution testing has been widely utilized for understanding the drug dissolution mechanism, predicting the bioavailability of the drug *in vivo*, evaluating the quality of drugs, and developing new formulations [8, 9]. Dissolution testing is also essential for the evaluation of generic drug consistency. Thus, a powerful dissolution method is important for the quality control of alfacalcidol tablets. Until now, there is no official monograph on the determination of the dissolution of alfacalcidol tablets.

UV and HPLC are commonly utilized to quantify the drug dissolution content, but none of the method is compatible with alfacalcidol tablets because of the weak ultraviolet absorption and influence of excipients. For compendia Apparatus 1 (basket) and Apparatus 2 (paddle), the volume of the dissolution medium may vary from 500 to 1000 mL [10]. Because the typical dosage of an alfacalcidol tablet is 0.25 *μ*g or 0.5 *μ*g, the final concentration of alfacalcidol after dissolution is 0.5 ng per milliliter or lower. Therefore, it could not be quantified by UV or HPLC methods. On the other hand, LC-MS has been reported as a highly sensitive, fast, and versatile method for quantification of vitamin D compounds in biological fluids [11, 12]. But, the application of LC-MS in directly characterizing alfacalcidol dissolution content is still confined due to poor ionization efficiency of API by electrospray ionization (ESI) source.

**Figure 1:**
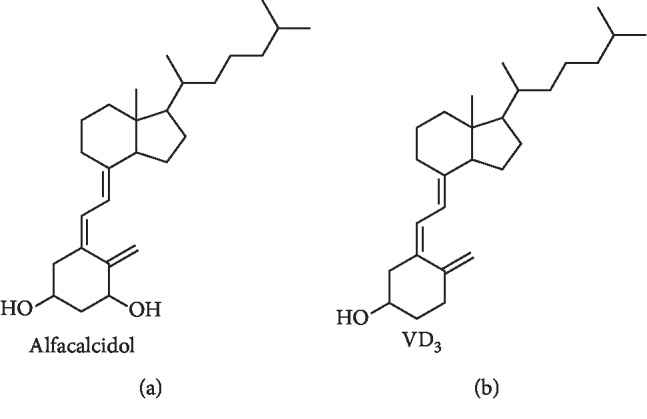
Structures of alfacalcidol and VD_3_ (IS).

It is reported that vitamin D in body fluids can be derivatized with Cookson reagents and then effectively quantified by LC-MS [3, 4, 11]. Different Cookson reagents have been developed and used to improve the sensitivity of vitamin D analysis [11, 12]. Because of the similar structure of Vitamin D_3_ (VD_3_) and alfacalcidol, we developed a new method to extract alfacalcidol from tablet dissolution tests (Figure 1). After derivatization with 4-phenyl-1, 2, 4-triazoline-3, 5-dione (PTAD) under ambient conditions, the derivatives were then accurately quantified by LC-MS using VD_3_ as the internal standard (IS).

## 2. Materials and Methods

### 2.1. Materials and Instruments

Alfacalcidol and VD_3_ were obtained from National Institutes for Food and Drug Control (Beijing, China). PTAD was purchased from Tokyo chemical industry (Tokyo, Japan). Ultrapure water was produced in-house by a Milli-Q water purification system (Millipore, Bedford, MA, USA). HPLC grade methanol, ethanol, dichloromethane, cyclohexane, ethyl acetate, and formic acid were from Fisher Scientific (Pittsburgh, USA). Alfacalcidol tablets (0.25 *μ*g or 0.5 *μ*g per tablet) were supplied by Yao Pharma (Chongqing, China). All reagents and solvents were used without further purification.

Dissolution experiment was carried out with dissolution apparatus RCZ-8MD from Tianjin TDTF Technology (Tianjin, China). A 1290 Infinity II UPLC Agilent connected to a 6470 series triple quadruple mass spectrometer system was used for LC-MS experiments (Santa Clara, CA, USA).

### 2.2. Methods

#### 2.2.1. Preparation of Samples

The stock standard solutions of alfacalcidol and VD_3_ (internal standard, IS) were prepared in ethanol as 0.1 *μ*g/mL and 1 *μ*g/mL, respectively. The working solutions were prepared by diluting the stock standard solution with dissolution medium (freshly prepared daily). Final working solutions contain 0.05–2 ng/mL of alfacalcidol and 1 *μ*g/mL of IS.

#### 2.2.2. Dissolution Experiment

Five-hundred milliliter (±1%) phosphate buffer (sodium dihydrogen phosphate) at pH 6.8 combined with 0.1% (w/v) sodium dodecyl sulfate (SDS) was used as the dissolution medium. Dissolution temperature was maintained at 37.0 ± 0.5°C, and the paddle apparatus (United States Pharmacopeia (USP) apparatus 2) was applied at the speed of 50 rpm. Five-milliliter sample solutions were withdrawn from the vessel with a glass syringe at 5, 15, 30, and 60 minutes interval, and the same volume of fresh dissolution medium was added to the vessels immediately after withdrawing. Samples were collected into a glass tube after filtration with a 0.45 *μ*m polyethersulfone (PES) membrane.

#### 2.2.3. Extraction SDS

Alfacalcidol samples or working samples (2 mL) were placed in glass centrifuge tubes. One milliliter of dichloromethane and 600 *μ*L of saturated saline solution were added. The solution was mixed thoroughly by vibration before being centrifuged at a speed of 2000 rpm for 3 minutes. After the organic layer was carefully removed, residual aqueous layer was further extracted with additional 1 mL dichloromethane. The organic phases were then combined and dried under nitrogen flow.

#### 2.2.4. Derivatization

PTAD (0.2 mg/mL) in ethyl acetate (50 *μ*L) was added to previously dried samples and kept at room temperature in dark. After 40 minutes, 50 *μ*L of ethanol was added to terminate the reaction. The final solution was dried with nitrogen, and the residue was redissolved in 0.1% formic acid-water and methanol in a ratio of 1 : 9 (v/v) (200 *μ*L) for LC-MS analysis.

#### 2.2.5. LC-MS Parameters

A Symmetry Shield RP18 column (3.0 mm × 150 mm, 3.5 *μ*m) with a precolumn was used for LC-MS at 40°C. The mobile phases were 0.1% formic acid-water and methanol in a ratio of 1 : 9 (v/v). The flow rate was 0.5 mL/min. An ESI source with positive-ion mode was used, and the parameters were as follows: sheath flow and temperature were 11 L/min and 350°C, respectively; gas flow and temperature were 11 L/min and 300°C, respectively; nozzle voltage was 500 V; nebulizer was 45 psi; and capillary voltage was 4000 V. High-purity nitrogen (>99.999%) was used as the collision gas in the multiple reaction monitoring (MRM) mode. Other MS parameters were as follows: alfacalcidol, fragmentation voltage: 170 V, collision energy: 10 V, 401.3 ⟶ 383.3 (m/z); alfacalcidol-PTAD, fragmentation voltage: 120 V, collision energy: 17 V, 576.4 ⟶ 314.1 (m/z); and IS-PTAD, fragmentation voltage: 100 V, collision energy: 13 V, 560.0 ⟶ 298.1 (m/z). MassHunter software (v 4.1, Agilent) was used for the data acquisition.

#### 2.2.6. Specificity and Calibration Curve

The dissolution medium which was pretreated and derivatized following procedures described in Sections 2.2.3 and 2.2.4 was injected to LC-MS as blank.

The dissolution medium spiked with 0.05, 0.1, 0.25, 0.5, 1, and 2 *μ*g/mL of alfacalcidol with 1 *μ*g/mL IS was pretreated and derivatized following procedures described in Sections 2.2.3 and 2.2.4. 10 *μ*L of derivatized solution was then subjected to LC-MS. The calibration curve was constructed by plotting the peak area ratios alfacalcidol-PTAD/IS-PTAD (*y*) versus the concentration of alfacalcidol (*x*).

#### 2.2.7. Assay Precision

The intra- and inter-assay precisions were achieved using two working solutions of alfacalcidol at 0.5 *μ*g/mL and 1 *μ*g/mL, each containing 1 *μ*g/mL of IS. The intra-assay was assessed by continuous injection of six times in one day and was finished within three days.

#### 2.2.8. Assay Accuracy

The assay accuracy solutions were prepared by dissolving alfacalcidol tablets in dissolution medium, and various amount of alfacalcidol containing IS was added. The recovery ratio (%) was defined as F/(F0 + A) × 100%, where F, F0, and A represents the concentration of alfacalcidol in the spiked sample, the concentration of alfacalcidol in the unspiked sample, and the spiked concentration.

#### 2.2.9. Stability

The stability of alfacalcidol solution was studied by determining 3 individual samples at 0.5 *μ*g/mL after 24 hours.

## 3. Results and Discussion

### 3.1. Dissolution Experiment

In order to develop a satisfactory dissolution testing method, the suitable composition of dissolution medium and volume which maintain sink conditions are necessary. Alfacalcidol is mainly absorbed in the small intestine [13]. Thus, phosphate buffer at pH 6.8 was used as the dissolution medium to simulate the environment of the small intestine. 0.1% (w/v) sodium dodecyl sulfate (SDS) was added into the dissolution medium to reduce the adsorption of alfacalcidol in the dissolution vessel or other containers. It is reported that potassium dihydrogen phosphate which was recommended in pharmacopeia for preparation of phosphate buffer (pH = 6.8) could possibly precipitate SDS. Sodium dihydrogen phosphate was used in buffer to avoid precipitation according to USP 1092 [10]. Thus, sodium phosphate was utilized to prepare pH 6.8 phosphate buffer in this study. No precipitation of SDS or phosphate salt was found during all the experiments.

### 3.2. Extraction and Derivatization

Since aqueous media are not compatible with Cookson reagent and SDS is harmful to the mass spectrometer, it is necessary to pretreat dissolution samples. Since alfacalcidol was readily soluble in dichloromethane [7], it is reported that vitamin D is soluble in cyclohexane [14], dichloromethane and cyclohexane mixture was first studied for liquid-liquid extraction. It was found that interfacial emulsification occurred during liquid-liquid extraction. Similar interfacial emulsification persists when cyclohexane was used as the extraction solvent. When dichloromethane was used alone, two clear layers could be achieved after adding 600 *μ*L saturated saline to emulsification and oscillation. Therefore, dichloromethane was chosen as a liquid-liquid extraction solvent.

After extraction, derivatization of alfacalcidol and IS with PTAD was achieved at room temperature based on previously reported derivatization of vitamin D [15, 16]. Various reaction time was investigated to achieve optimal efficacy. Results showed that more than 98% of alfacalcidol was consumed after 40 minutes. Thus, 40 minutes was used as the reaction time.

### 3.3. LC-MS Analysis

In MS quantitative analysis, isotope-labeled compounds are considered as the ideal IS. However, for many APIs, the isotope-labeled IS was very expensive or difficult to access. Nonisotopic substances which have similar structure to the analyte as IS gained great interests [17]. Here, VD_3_ which is one hydroxyl group less than alfacalcidol was used as the IS. Both alfacalcidol and IS could react with PTAD to generate a six-membered ring. VD_3_ used as the IS in this study was much cheaper than radio-labeled alfacalcidol.

**Figure 2:**
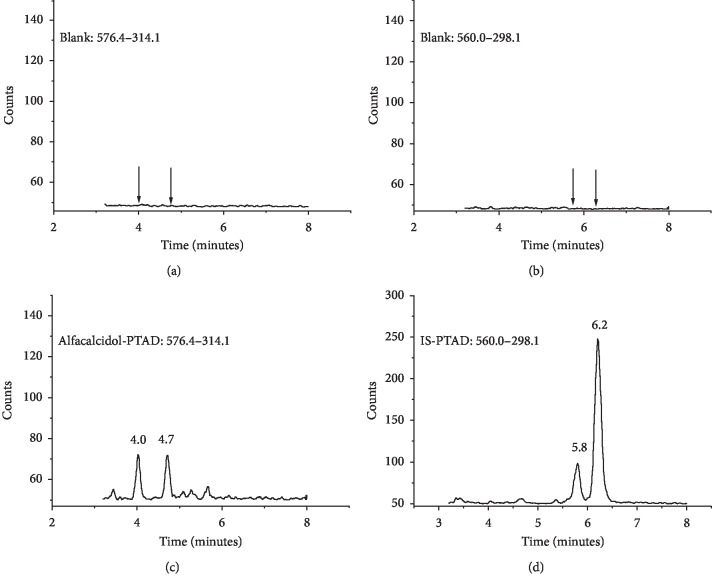
MRM chromatograms. (a) Blank (576.4–314.1); (b) blank (560.0–298.1); (c) 0.05 *μ*g/mL alfacalcidol–PTAD; (d) 1 *μ*g/mL IS-PTAD.

**Figure 3:**
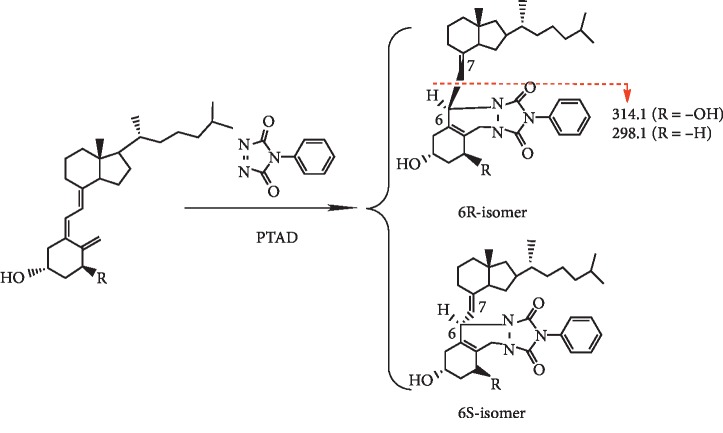
Derivatization of alfacalcidol (R = OH) and IS (R = H) with PTAD.

Two eluting peaks were found in the total ion chromatography (TIC) of alfacalcidol-PTAD and IS-PTAD (Figure 2). According to the reports, the mixture of IS-PTAD derivatives included 6S and 6R isomers because the *α* and *β* sides of vitamin D compounds interacted with PTAD in the hetero Diels–Alder reaction [15]. Thus, the two peaks from alfacalcidol-PTAD were also caused by 6S and 6R isomers (Figure 3). The ratio of alfacalcidol-PTAD isomers was 0.8–1.2, and the ratio of IS-PTAD isomers was around 0.2 (6R/6S). The 6S-isomer of vitamin D derivative was superior to the 6R-isomer because of the steric hindrance of the 3*β*-hydroxy group in the A-ring of vitamin D [18]. Since there are two hydroxyl groups in alfacalcidol, the space hinderance of *α* and *β* sides are similar. The ratio of 6S and 6R is around one which is consistent to the peak area in TIC.

Alfacalcidol, alfacalcidol-PTAD, and IS-PTAD were analyzed with MRM mode. The ion pairs were as follows: alfacalcidol (401.3 ⟶ 383.3), alfacalcidol-PTAD (576.4 ⟶ 314.1), and IS-PTAD (560.0 ⟶ 298.1). The daughter ions of alfacalcidol-PTAD (314.1) and IS-PTAD (298.1) were from the cleavage of C6-7 bond during MS analysis (Figure 3). It is consistent with the structure proposed in a previous report [19]. The limit of quantification (LOQ) of alfacalcidol-PTAD was 0.05 *μ*g/mL (S/N = 10), and the good reproducibility of LOQ was achieved by repeating 5 injections of samples at 0.05 *μ*g/ml. Meanwhile, the LOD of the alfacalcidol without any pretreatment was around 1 *μ*g/mL after extraction and redissolution. The LOD of alfacalcidol was improved more than 100 times after chemical derivatization. The LOQ of alfacalcidol-PTAD was comparable to the LOQ of 25(OH)D_3_-PTAD in the literature (0.05 *μ*g/mL versus 0.025–0.3 *μ*g/mL) [16, 20, 21].

### 3.4. Method Validation

#### 3.4.1. Specificity and Calibration Curve

**Figure 4:**
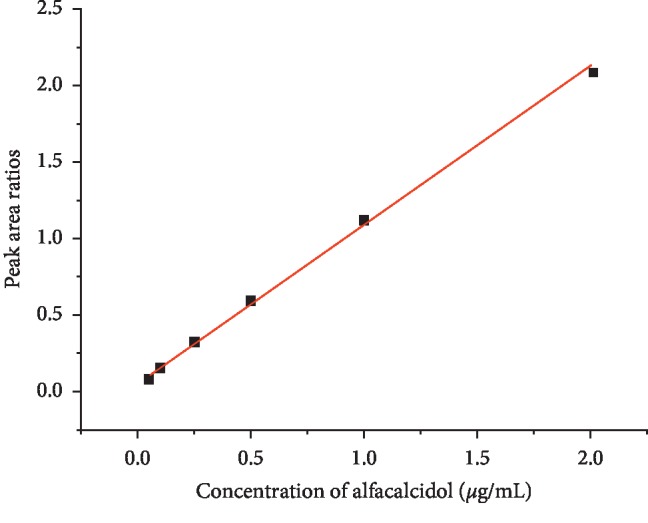
Calibration curve of 0.05–2 *μ*g/mL alfacalcidol.

The dissolution medium showed no interference and the calibration curve was *y* = 1.04177*x* + 0.05124, as can be seen in Figure 4, it shows good linearity ranging from 0.05 *μ*g/mL to 2 *μ*g/mL (*r*^2^ > 0.99, all the results were from 3 injections).

#### 3.4.2. Assay Precision and Assay Accuracy

Intraday and interday of precisions at 0.5 *μ*g/mL and 1 *μ*g/mL were less than 3.3% and 7.9%, respectively (Table 1). The assay accuracy was assessed by dissolution solutions (*n* = 3) spiked with three different concentrations of alfacalcidol (Table 2). The recovery of 0.4 *μ*g/mL, 0.5 *μ*g/mL, and 0.6 *μ*g/mL level were 100.03 ± 9.77%, 104.31 ± 4.53%, and 95.30 ± 4.16%, respectively. The method renders high reproducibility and accuracy in analyzing alfacalcidol dissolution content.

#### 3.4.3. Stability

Alfacalcidol solution was stable at room temperature in dark after 24 hours.

### 3.5. Actual Samples Dissolution Detection

**Figure 5:**
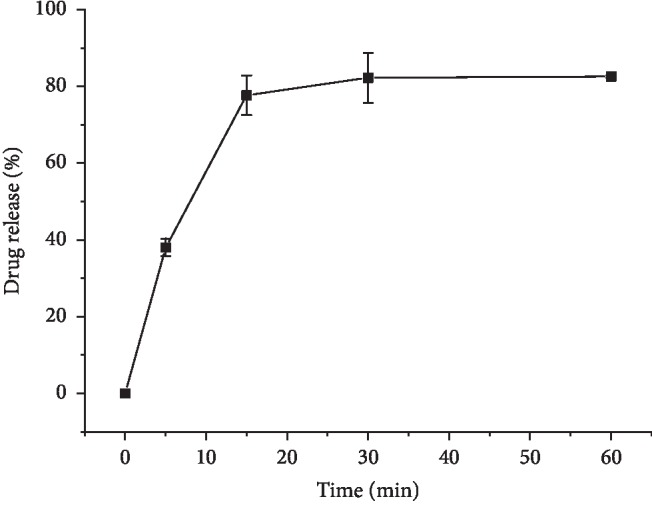
Dissolution curve of alfacalcidol finished product (0.25 *μ*g per tablet).

Dissolution of actual alfacalcidol tablets (0.25 *μ*g or 0.5 *μ*g per tablet) was performed in 500 mL dissolution media following the method described in Section 3.1. After 5–60 minutes incubation, the dissolution solution was extracted, derivatized, and analyzed by LC-MS. Final data showed that alfacalcidol dissolution increased to around 75% in 15 minutes and reached platform after about 30 minutes (Figure 5). The signal-to-noise ratio (S/N) was about 18 at 0.2 *μ*g/mL (first dissolution point at 5 minutes) and fulfill the quantitation requirement. SDS and phosphate in the dissolution media was effectively removed by extraction and did not interfere with mass spectrometry tests.

## 4. Conclusions

Establishment of alfacalcidol tablet dissolution is challenging due to the difficulty of measuring low-dosage alfacalcidol in dissolution media. Here, a chemical derivatization combined with the LC-MS method has been developed and validated for quantification of alfacalcidol tablets dissolution content. Compared with the nonderivatized LC-MS method, the LOD of new method increased 100 folds (from 1 *μ*g/mL to 0.01 *μ*g/mL). The method reported here has high sensitivity, precision, and accuracy. It could be widely used in the quality control of small-dosage solid pharmaceuticals.

## Figures and Tables

**Table 1:** The assay precision of the method (*n* number of determinations at each concentration).

Nominal concentration (*μ*g/mL)	Intraday assay (*n* = 6)		Interday assay (*n* = 3)	
	Calculated concentration (*μ*g/mL)	RSD%	Calculated concentration (*μ*g/mL)	RSD%
0.5	0.55 ± 0.02	2.5	0.52 ± 0.04	4.8
1.0	1.15 ± 0.07	3.3	1.01 ± 0.11	7.9

**Table 2:** The assay accuracy of the method (*n* number of determinations at each concentration).

	Nominal concentration (ng/mL) (*n* = 3)	Measured concentration (ng/mL)	RE%	RSD%
1	0.79	0.79 ± 0.07	100.03 ± 9.77	7.1
2	0.89	0.92 ± 0.04	104.31 ± 4.53	3.3
3	0.99	0.92 ± 0.03	95.30 ± 4.16	2.4

## Data Availability

The data used to support the results of this study are included within the article. Any further information is available from the authors upon request.
